# Predictors of hospital readmission rate in geriatric patients

**DOI:** 10.1007/s40520-023-02664-9

**Published:** 2024-02-07

**Authors:** Arianna Bortolani, Francesco Fantin, Anna Giani, Alessandra Zivelonghi, Bruno Pernice, Elena Bortolazzi, Silvia Urbani, Elena Zoico, Rocco Micciolo, Mauro Zamboni

**Affiliations:** 1 Section of Geriatric Medicine, Department of Surgery, Dentistry, Pediatric and Gynecology, University of Verona, 37126 Verona, Italy; 2 Section of Geriatric Medicine, Centre for Medical Sciences - CISMed, Department of Psychology and Cognitive Science, University of Trento, Rovereto (TN), Italy; 3 Section of Geriatric Medicine, Department of Medicine, University of Verona, Verona, Italy; 4 Centre for Medical Sciences, Department of Psychology and Cognitive Sciences, University of Trento, Trento, Italy

**Keywords:** Hospital readmissions, Readmission risk, Predictors, Comprehensive geriatric assessment

## Abstract

**Background:**

Hospital readmissions among older adults are associated with progressive functional worsening, increased institutionalization and mortality.

**Aim:**

Identify the main predictors of readmission in older adults.

**Methods:**

We examined readmission predictors in 777 hospitalized subjects (mean age 84.40 ± 6.77 years) assessed with Comprehensive Geriatric Assessment (CGA), clinical, anthropometric and biochemical evaluations. Comorbidity burden was estimated by Charlson Comorbidity Index (CCI). Median follow-up was 365 days.

**Results:**

358 patients (46.1%) had a second admission within 365 days of discharge. Estimated probability of having a second admission was 0.119 (95%C.I. 0.095–0.141), 0.158 (95%C.I. 0.131–0.183), and 0.496 (95%C.I. 0.458–0.532) at 21, 30 and 356 days, respectively. Main predictors of readmission at 1 year were length of stay (LOS) > 14 days (*p* < 0.001), albumin level < 30 g/l (*p* 0.018), values of glomerular filtration rate (eGFR) < 40 ml/min (*p* < 0.001), systolic blood pressure < 115 mmHg (*p* < 0.001), CCI ≥ 6 (*p* < 0.001), and cardiovascular diagnoses. When the joint effects of selected prognostic variables were accounted for, LOS > 14 days, worse renal function, systolic blood pressure < 115 mmHg, higher comorbidity burden remained independently associated with higher readmission risk.

**Discussion:**

Selected predictors are associated with higher readmission risk, and the relationship evolves with time.

**Conclusions:**

This study highlights the importance of performing an accurate CGA, since defined domains and variables contained in the CGA (i.e., LOS, lower albumin and systolic blood pressure, poor renal function, and greater comorbidity burden), when combined altogether, may offer a valid tool to identify the most fragile patients with clinical and functional impairment enhancing their risk of unplanned early and late readmission.

## Introduction

Non-elective hospital readmissions after discharge from acute episodes of illness represent a significant issue among older adults. Recurrent re-hospitalizations are associated with progressive worsening of functional state, increased risk of institutionalization and increased mortality, along with higher costs for health systems.

Recently, the research has identified several predictors of early and late re-hospitalization. Demographic characteristics, such as age, sex, race [1–3], but moreover clinical variables, were identified as predictors of readmission. Among clinical data, the main diagnosis, either surgical or medical [4], and a variety of specific diagnoses, such as coronary artery disease, congestive heart failure, chronic obstructive pulmonary disease (COPD) [5], malignancy, new onset of dementia [6], diabetes, and chronic kidney disease [7] were related to readmission.⁠ In a selected population of older heart failure patients, systolic blood pressure below 120 mmHg was associated with increased risk of early re-hospitalization [4]. In addition to specific diagnoses, the burden of comorbidities itself [8,9] and consequent polypharmacy [10,11] were related to readmission too. Laboratory findings, such as impaired renal function [12,13] and low albumin level (< 30 g/l) [14,15] were identified as predictors of readmission. Inadequate nutritional status [16–18] was also found to be associated with increased risk of re-hospitalization. Frailty was found to increase the risk of early readmission, meanwhile a low degree of independence in Instrumental Activity of Daily Living (IADL) and pre-existing index hospitalization, were also found to increase readmission rate at 60–90 days [19].

Although several demographic, clinical, laboratory, functional variables have been associated in different studies with higher risk of readmission of older patients, an extensive evaluation and comparison of the effects of these variables in the same study population that employs the Comprehensive Geriatric Asessment (CGA), is still lacking.

The main aim of our study was to identify the main predictors of readmission at 1 year in 777 older adults hospitalized in a geriatric ward, who were evaluated with the CGA.

## Materials and methods

### Study population

**Fig. 1:**
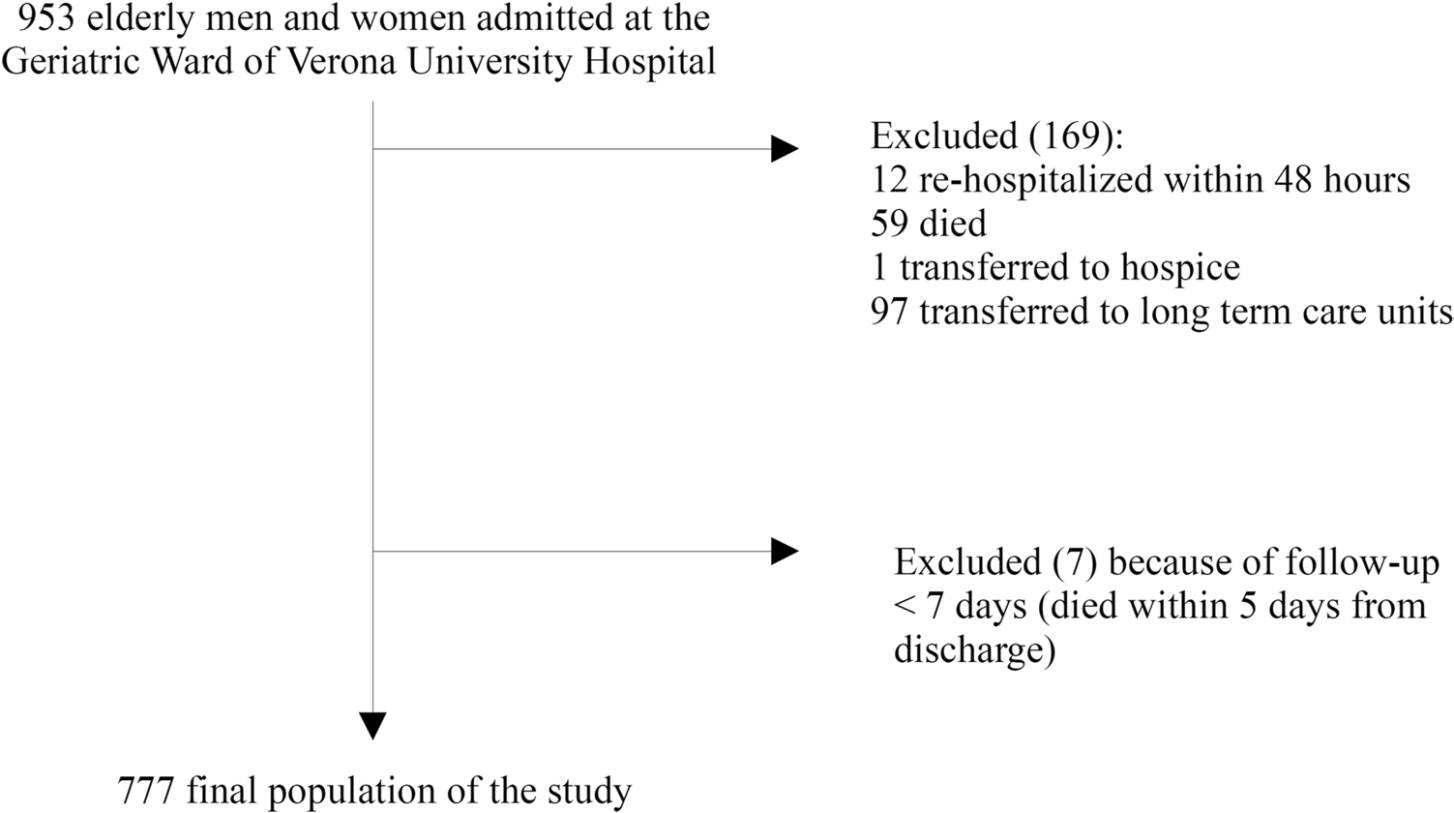
Study population flowchart

The initial study population consists of all the 953 patients consecutively admitted to the Geriatric Unit of Verona University Hospital from Sep 2018 to Dec 2019. The 97% of the patients were admitted after initial assessment in the Emergency Department of the Hospital. The clinical management after discharge was ensured for all the patients by the General care Practitioner (family doctor). From a total of 953 patients, 169 were excluded from the study sample, because 12 were re-hospitalized within 48 h, 59 died during hospital stay, 1 was transferred to hospice, 97 to long-term care units, 7 died at home within 5 days from discharge. Therefore, a total of 777 subjects were evaluated. The median follow-up was 365 days. (Fig. 1).

The following inclusion criteria were considered: age 65 years and above; written informed consent with patient’s signature if patient is capable of participating in a structured interview and understand the objective of the study, otherwise with the caregiver’s signature. Patients transferred to other hospital wards and those discharged to long-term care facilities or rehabilitation units, were excluded from the study.

The study was approved by the Ethics Committee of the hospital, and it was conducted in accordance with the latest revision of the Helsinki Declaration as well as the Oviedo Declaration.

A written consent to participate to the study was signed by all the subjects or by the proxy care giver in patients unable to provide consent.

### Comprehensive geriatric assessment

All the patients underwent a complete clinical evaluation, comprehensive of detailed clinical history, pathological conditions, previous admissions and drug regimen. Medical care was offered to participants according to national and international standards of clinical practice.

Body weight and height were assessed in all the subjects and Body Mass Index (BMI) was calculated as weight (Kg)/height^2^(m^2^) [20] at admission. Biochemical evaluation was obtained in all the subjects with full panel of laboratory tests as per routine clinical practice. Among the laboratory results, only albumin level and glomerular filtration rate (eGFR) (obtained by Cockroft-Gault equation) were included in the analysis. Blood pressure was evaluated in all the subjects, lying in a clinostatic position after adequate rest and repeated three times. The average values were utilized for the analysis.

Items of CGA were performed during the first 24–48 h, or as soon as possible when the clinical conditions of the patient were critical at admission time. Reported pre-admission abilities referred to the last 1 week before admission, either self-reported by the patient or reported by the care giver: Activities of Daily Living (ADL) were assessed according to Katz [21], IADL according to Lawton [22],^.^ Barthel Index [23] was used to assess functional independence in the domains of daily and self-care activities and mobility. Cognitive function was evaluated using the ‘Mini-Mental State Evaluation’ (MMSE) [24]. Depression was investigated through a validated shorter version of the Geriatric Depression Scale (GDS-15 items), a self-report instrument tailored to screen depression in older adults [25]. For patients with severe cognitive impairment, compromising the ability to self-report symptoms or activities, CGA was obtained with the contribution of the care giver. Self-reported scales, not suitable for being answered by the care giver (e.g., GDS), were recorded as ‘not applicable’. Comorbidity was evaluated utilizing Charlson Comorbidity Index (CCI) [26]. Nutritional status was evaluated with the Mini-Nutritional Assessment (MNA) form [27].

A follow-up was scheduled after discharge in order to investigate patient’s conditions, re-hospitalizations either in the same hospital or at different health facilities, and death. A structured telephone call conducted by the study investigators to either the patient or the caregiver was performed at 48 h after discharge, then at 1 week, thereafter weekly for the first month and monthly for the following 11 months.

### Statistical analysis

Data are shown as mean, standard deviation, median, lower and upper quartile. Mann–Whitney’s *U* test was used to compare medians between male and female subjects.

The main subject of the analysis was the time elapsed between the day of discharge and the day on which a patient was admitted a second time. The follow-up period was extended to 365 days. The probability of re-hospitalization has been estimated with the Kaplan and Meier method [28], while two or more “readmission curves” were compared using the log-rank test [29]. The combined effect of several variables on the probability of re-hospitalization was evaluated employing the Cox model [30]. To evaluate the performance of the final model, employing the regression coefficients estimated by the Cox model, a score was calculated for each subject quantifying his/her risk of re-admission in the 365 days following discharge. This score was calculated in such a way that a value of zero identifies an overall “average” risk. Therefore, subjects with a negative score should have a risk lower than average, while the opposite applies to subjects with a positive score. Two measures of effect size were employed to quantify the impact of the different variables on the chance of readmission: the hazard ratio (HR), estimated by means of the Cox model, and the expected absolute number of readmitted subjects in the higher risk group (or groups) of the considered variables with respect to the lower risk group. Expected number of readmissions were calculated applying the daily rate of readmission estimated on subjects belonging to the lower risk group to the person-days at risk of subjects belonging to the higher risk group (or groups). The level of statistical significance was set at *p* < 0.05.

## Results

The characteristics of the study population are shown in Table 1.

**Table 1:** Characteristics of the study population

	Male (*n* 386)					Female (*n* 391)						Missing (overall)
	Mean	s.d.	Median	I q.le	III q.le	Mean	s.d.	Median	I q.le	III q.le	*p*	
Age(years)	83.42	6.85	83.00	78.00	89.00	85.36	6.56	86.00	81.00	90.00	< 0.001	0
Weight (kg)	73.45	14.21	72.83	63.15	80.90	63.67	13.95	62.50	54.30	72.20	< 0.001	32
Hb (g/L)	115.01	21.22	115.00	101.00	130.00	114.41	19.45	116.00	102.00	128.00	0.796	0
RBC (*10^12^/L)	3.90	0.72	3.96	3.44	4.34	4.00	0.67	4.03	3.51	4.41	0.063	16
WBC(*10^9^/L)	9.58	6.60	8.27	6.36	11.20	10.02	6.51	8.70	6.64	11.30	0.173	16
Platelets (*10^9^/L)	218.65	97.30	200.00	154.00	265.00	243.21	96.75	233.00	177.00	295.00	< 0.001	18
Creatinin (umol/L)	123.12	80.51	100.00	78.00	141.00	94.93	48.53	82.00	63.00	109.00	< 0.001	13
eGFR (ml/min)	51.75	24.51	48.30	32.77	66.72	45.76	22.72	41.46	29.62	57.59	< 0.001	41
Albumin (g/L)	31.35	5.22	31.45	27.90	35.10	31.35	5.75	31.70	27.60	35.60	0.688	11
CRP (mg/L)	73.99	72.50	53.00	18.00	107.00	67.86	74.77	42.00	15.00	91.00	0.115	95
SBP (mmHg)	127.35	19.44	125.00	115.00	140.00	129.36	19.60	130.00	115.00	140.00	0.117	1
DBP (mmHg)	71.71	10.23	70.00	65.00	80.00	72.79	10.28	70.00	65.00	80.00	0.224	1
MAP (mmHg)	90.26	12.01	90.00	83.33	96.66	91.65	12.22	91.67	83.33	100.00	0.156	1
PP (mmHg)	55.64	15.23	50.00	45.00	65.00	56.57	14.89	55.00	45.00	65.00	0.307	1
HR (bpm)	78.82	15.69	78.00	68.00	88.00	83.79	15.97	80.00	74.00	90.00	< 0.001	2
LOS (days)	11.66	8.65	9.00	7.00	14.00	10.98	6.53	9.00	7.00	13.00	0.932	0
ADL	4.45	2.15	6.00	3.00	6.00	3.64	2.43	5.00	1.00	6.00	< 0.001	2
IADL	4.24	3.06	5.00	1.00	7.00	3.55	3.20	3.00	0.00	7.00	0.002	2
MMSE	16.22	10.53	19.30	5.00	26.70	13.60	10.13	5.00	5.00	24.40	0.001	0
MNA	15.40	4.64	14.00	12.00	19.00	15.74	4.41	14.00	13.00	19.00	0.129	33
GDS	2.51	2.42	2.00	1.00	4.00	3.50	2.68	3.00	2.00	5.00	< 0.001	142
BI	52.60	34.08	55.00	20.00	85.00	41.71	30.82	40.00	10.00	65.00	< 0.001	0
CCI	6.19	2.16	6.00	5.00	8.00	5.86	1.75	6.00	5.00	7.00	0.103	0

*p* p-value of the Mann–Whitney’s *U* test. *Hb *Haemoglobin; *RBC* red blood cell; *WBC *white blood cell; *eGFR *glomerular filtration rate; *CRP *C Reactive Protein; *MAP *mean arterial pressure; *PP *pulse pressure; *HR *heart rate; *bpm *beats per minute; *ADL *Activities of Daily Living; *IADL *Instrumental Activities of Daily Living; *MMSE *Mini-Mental State Examination; *MNA *Mini-Nutritional Assessment; *GDS* Geriatric Depression Scale; *BI *Barthel Index;* CCI *Charlson Comorbidity Index

Women were significantly older than men and showed lower body weight, lower values of creatinine and eGFR compared with men.

Women showed also significantly lower scores of ADL, IADL, Barthel index, MMSE and higher scores of GDS compared with men.

More than one patient out of two had serious cognitive impairment according to MMSE (54.6%) or malnutrition according to MNA (63%).

Average length of stay was not significantly different between women and men.

**Fig. 2:**
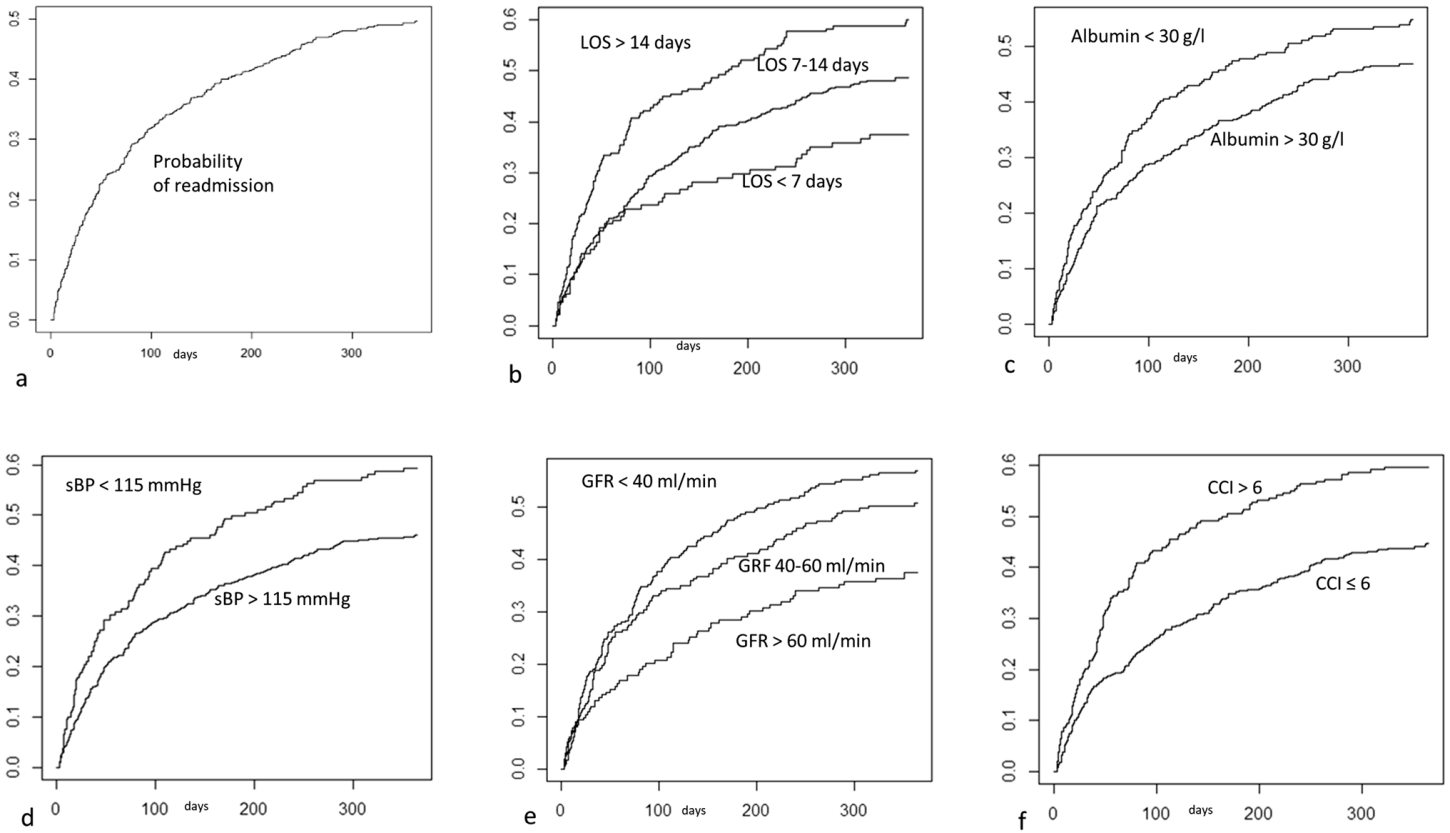
Hospital readmission risk according to different variables. **a** probability of readmission; **b** 1 year re-admission risk according to length of stay; **c** 1 year re-admission risk according to albumin level; **d** 1 year re-admission risk according to systolic blood pressure; **e** 1 year re-admission risk according to eGFR; **f** 1 year re-admission risk according to Charlson comorbidity index

The probability of having a second admission according to the time elapsed from the date of discharge was estimated employing the Kaplan–Meier method [28] and is shown in Fig. 2a. Overall, after 365 days of follow-up time, the estimated probability of having a second admission was 0.496 (95% C.I.: 0.458–0.532); therefore, the median readmission time cannot be estimated, even if it is likely to be just over 365 days. The estimated probability of having a second admission was 0.119 after 3 weeks (95% C.I.: 0.095–0.141) and 0.158 after 30 days (95% C.I: 0.131–0.183). In total, 358 patients had a second admission within 365 days of discharge, while the total observation time was 161,287 patient/days; therefore, the overall average rate of re-admission was 358/161287 = 2.22 per thousand patient/days, or, referring to an “average” month of 30.4 days, 6.75 percent patient/months (i.e., of 100 patients discharged, about 7 are admitted on average in a month). However, the risk of re-admission is not constant over time: it is higher immediately after discharge and then decreases relatively quickly. In particular, the re-admission rate within 4 weeks of discharge is 5.79 per thousand patient/days (more than 2 and a half times the general average); between the fifth and eighth week it falls to 4.20 and between the ninth and twelfth, it is 2.63. Starting from the 17th week, the rate decreases steadily below the general average value. After the 36th week, the rate drops below half of the total.

The association between the risk of readmission and selected variables was evaluated by comparing “readmission curves” in sub-groups of patients by means of the log-rank test. As far as length of stay is concerned, patients were divided into three groups, depending on whether the length of stay lasted 7 days or less (144 patients; group A), between 8 and 14 days (412 patients; group B) or more than 14 days (221 patients; group C); a highly significant difference in the probability of readmission was found (*p* < 0.001; Fig. 2b). After 180 days of discharge, half of patients of group C were readmitted, whilst more than 60% of patients of group B and over 70% of patients of group A did not have a second admission. Overall, group B showed a hazard for readmission that was about 1.4 times that of group A, while group C showed a hazard for readmission that was about double than that of group A. In absolute values, 119 out of 221 patients of group C were readmitted while 61 were expected; on the other hand, 188 out of 412 patients of group B were readmitted while 137 were expected.

When considering albumin and adopting a cut off of 30 g/l, patients with low albumin levels (*n* = 300) showed a significantly greater probability of re-hospitalization (*p* = 0.018) when compared with patients with higher albumin levels (*n* = 466) (Fig. 2c). The median time of readmission in patients with low albumin was 240 days (i.e., the probability of readmission within 240 days after discharge was 50%), while in the same timeframe, more than 58% of patients with high albumin were not readmitted. Overall, patients with low albumin levels showed a hazard for readmission that was about 1.3 times that of other patients. In absolute values, 148 out of 300 patients with low albumin levels were readmitted while 114 were expected.

When considering the Barthel index at discharge (with a threshold of 60; corresponding to 465 and 312 patients below and above the threshold, respectively), there was no statistically significant difference between the readmission curves in patients with low and high care needs (*p* = 0.176).

As far as systolic blood pressure at the time of admission is concerned, subjects were divided into two groups, and a threshold of 115 mmHg was adopted (211 and 565 subjects, respectively, below 115 and above 115 mmHg). A highly significant difference in the probability of re-hospitalization was found (*p* < 0.001), with a higher risk in patients with lower systolic blood pressure (Fig. 2d). The median time of readmission in this group of patients was 191 days, while in the same timeframe, more than 60% of the patients with higher blood pressure were not readmitted. Overall, patients with low systolic blood pressure showed a hazard for readmission that was about 1.5 times than that of other patients. In absolute values, 113 out of 211 patients with low systolic blood pressure were readmitted while 76 were expected.

A statistically significant difference in the probability of re-hospitalization was observed by subdividing the study sample into three groups of creatinine clearance, with the following cut offs: 40 and 60 ml/min. The 3 groups included 317, 226 and 193 patients for ranges of creatinine clearance below 40, between 40 and 60 and above 60 ml/min, respectively (Fig. 2e). After 209 days of discharge, half of patients with clearance below or equal to 40 were readmitted. After 315 days of discharge, half of patients with clearance between 40 (excluded) and 60 (included) were readmitted, whereas almost 64% of patients with clearance above 60 did not have a second admission. Overall, patients with clearance below or equal to 40 showed a hazard for readmission that was about 1.8 times than that of patients with clearance above 60, while patients with clearance between 40 and 60 showed a hazard for readmission that was about 1.2 times than that of patients with clearance above 60. In absolute values, 161 out of 317 patients with clearance below or equal to 40 were readmitted while 90 were expected; on the other hand, 110 out of 226 patients with clearance between 40 and 60 were readmitted while 73 were expected.

Comorbidity was evaluated with the Charlson Index and subjects were categorized into two groups, according to a score of equal or less than 6 (low comorbidity, 509 patients) or higher than 6 (high comorbidity, 268 patients); the two groups showed a highly significant difference in the probability of readmission (*p* < 0.001) (Fig. 2f). At 164 days after discharge, half of the patients in the high comorbidity group (CCI > 6) were readmitted, meanwhile 66% of the patients with a Charlson score lower than 6 were not. Overall, patients in the high comorbidity group showed a hazard for readmission that was about 1.6 times that of other patients. In absolute values, 144 out of 268 patients in the high comorbidity group were readmitted while 89 were expected.

The probability of readmission according to diagnoses was then tested. Those with a cardiovascular diagnosis (*n* = 243), were at significant greater risk of re-hospitalization (133 patients were readmitted within the follow-up period). After 230 days of discharge, half of the patients with a cardiovascular diagnosis were readmitted. For all the other considered diagnostic categories (infectious diseases, *n* = 139; respiratory failure, *n* = 169; dementia/cerebropathy, *n* = 32; anemia, *n* = 67; other diagnoses, *n* = 127) the risk of readmission was lower than that of patients with a cardiovascular diagnosis. Overall, patients with a cardiovascular diagnosis showed a hazard for readmission that was about 1.3 times than that of other patients. In absolute values, 133 out of 243 patients with a cardiovascular diagnosis were readmitted while 100 were expected.

The combined effect of the previously analyzed variables on the risk of readmission was assessed using the Cox model. Due to some missing data (no creatinine clearance was recorded for 41 of the 777 patients), analyses were carried out on a subset of 736 subjects with all the information available.

The starting model included all the following variables (coded as described above): length of stay, albumin, Barthel index, systolic blood pressure (SBP), creatinine clearance and CCI. From this model, Barthel index and albumin level were progressively removed, meanwhile the remaining four above-mentioned variables confirmed an independent and significant association with the risk of readmission.

Specifically, the final model showed a higher risk of re-hospitalization for: length of stay longer than 14 days, compared to length of stay lower than 8 days (HR 1.89; 95% CI 1.34–2.66); systolic blood pressure on admission equal or lower to 115 mmHg compared with higher than 115 mmHg (HR 1.38; 95% CI 1.10–1.74); and creatinine clearance with eGFR < 40 ml/min compared to creatinine clearance > 60 ml/min (HR 1.64; 95% CI 1.23–2.18), CCI > 6 (HR 1.44; 95% CI 1.16–1.80).

When the diagnosis was added to this model, all the above-mentioned variables still remained significantly and independently associated with the risk of re-admission.

To evaluate the performance of the final model i.e. to assess whether the variables selected were able, taken collectively, of identifying patients at different risk, a score was calculated, using the coefficients estimated by the Cox model, which included length of stay, SBP, creatinine clearance and CCI as prognostic variables (Table 2). These scores (ranging between − 0.870 and 0.947) were calculated in such a way that a value of zero identifies an overall “average” risk. If the final model performs well, subjects with a negative score should have a risk lower than average, while the opposite applies to subjects with a positive score.

**Table 2:** Cox Regression model

Results of the Cox regression model				
Variable	Category	Estimate	Standard error	*p*
Length of stay	1–7 days	0.000	−	
Length of stay	8–14 days	0.268	0.166	0.106
Length of stay	> 14 days	0.637	0.175	< 0.001
Systolic blood pressure	< 115 mm Hg	0.000	−	
Systolic blood pressure	> 115 mm Hg	− 0.322	0.117	0.006
Creatinine clearance	< 40 ml/min	0.000	−	
Creatinine clearance	40−60 ml/min	− 0.121	0.126	0.336
Creatinine clearance	> 60 ml/min	− 0.493	0.146	< 0.001
Charlson index	0−6	0.000	−	
Charlson index	> 6	0.366	0.113	0.001

To evaluate the performance of the final model in identifying subjects at different risk of readmission, three approximately equal-sized groups were considered on the basis of two score thresholds symmetrical to zero: patients with values below − 0.2 (group 1: 263 subjects, expected to have the lowest risk of readmission), patients with a score between − 0.2 and 0.2 (group 2: 228 subjects, expected to have an intermediate risk), and patients with a score above 0.2 (group 3: 245 subjects, expected to have the highest risk).

**Fig. 3:**
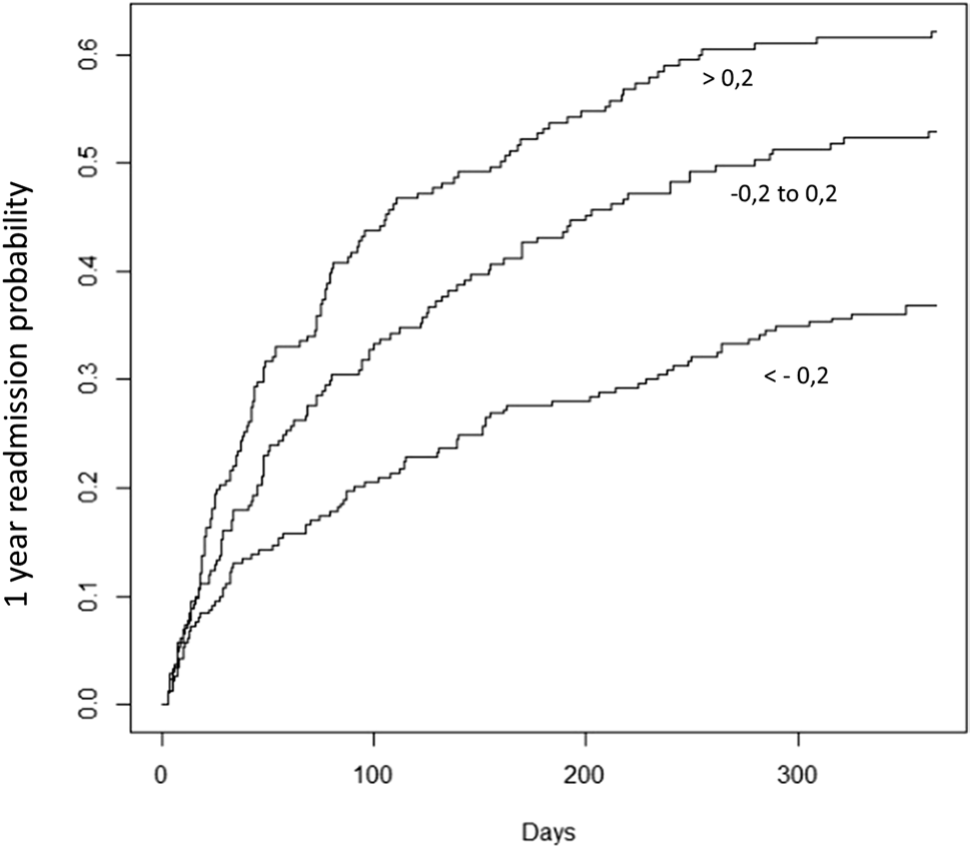
Probability of readmission at 1 year according to the score group. Three approximately equal-sized groups were considered on the basis of two thresholds symmetrical to zero: patients with values below − 0.2 (*n* = 263 subjects, lowest readmission risk), patients with a score between − 0.2 and 0.2 (*n* = 228, intermediate risk), and patients with a score above 0.2 (*n* = 245, highest risk)

Figure 3 shows the probability of re-hospitalization estimated in these three groups. Half of group 3 patients were readmitted within 160 days of discharge, while for group 2 patients the median time was 280 days. At this date, about 2/3 of the subjects in group 1 have not had a second admission yet. After 365 days of follow-up, the chances of readmission in the three groups are 0.378, 0.471 and 0.631, respectively. With regard to the median readmission time, group 2 has, compared to group 3, about four more months of “survival”. The median readmission time in group 1 is higher than 365 days, so, compared to group 2, group 1 has more than 85 days of “survival” and, compared to group 3, it has more than 205 days of “survival”.

A further comparison can be made considering for example a probability of readmission of 0.333. In this case, 1/3 of group 1 patients were readmitted within approximately 264 days, 1/3 of group 2 patients were readmitted within approximately 100 days, and 1/3 of group 3 patients were readmitted within approximately 54 days. Therefore, in this case, group 2 has, compared to group 3, 46 days “free from readmission” and group 1 has, compared to group 3, 210 days “free from readmission”. Overall, group 3 showed a hazard for readmission that was about 2.2 times that of group 1, while group 2 showed a hazard for readmission that was about 1.5 times that of group 1. In absolute values, 134 out of 245 patients of group 3 were readmitted while 62 were expected; on the other hand, 112 out of 228 patients of group 2 were readmitted while 68 were expected.

## Discussion

The present study investigated the main predictors of unplanned re-hospitalization within a timeframe of 365 days after discharge, in a cohort of 777 older adults consecutively admitted to an acute Geriatric Care Unit and evaluated with CGA. Though CGA is a pillar of geriatric evaluation and management, its extensive utilization in retrospective study designs is not always applicable; conversely, due to the prospective design of our study and due to the focus on CGA, we could provide a comprehensive characterization of the study patients and very few data and variable are missing. In the study cohort, hospital readmission was not constant over time ranging from 15% at 30 days, to 46.1% at 1 year. The risk of readmission was associated with longer length of stay (> 14 days), lower albumin level (< 30 g/dl), lower creatinine clearance (< 40 ml/min), a value of systolic BP < 115 mmHg, higher comorbidity (CCI > 6) and by a cardiovascular diagnosis.

Readmission rate at 30 days was investigated in several previously published papers, with heterogeneous populations and results. Our finding of a 30-day readmission rate of 15% is in line with other studies performed in acute settings [7,12,31]. However, a higher readmission rate (about 19%) was observed by other authors [3,18,32] who studied samples that differed in setting, age and comorbidity. Furthermore, we observed a 1-year readmission rate of 46.1%, in line with some, but not all, studies, mainly due to the different study design. In fact, Visade et al. [33], in their study of 3,081 patients aged 75 and above admitted to a French acute geriatric unit, used a similar design to our study and found a re-hospitalization rate at 1 year of 49.7%, whilst Sganga [17], with 921 patients, found a lower re-hospitalization rate at 1 year (30.4%), after excluding critical patients with a cancer diagnosis or severe malnutrition from the population. A higher re-hospitalization rate at 1 year (56.1%) was found by Jencks [32] in adult Medicare beneficiaries, with mixed medical and surgical conditions.

However, in our study, the highest readmission risk (50% at 180 days) was found in patients with length of stay (LOS) longer than 14 days. Meanwhile, for the central LOS group (between 8 and 14 days), risk of readmission begins to differentiate significantly from the lowest LOS group (7 days and below), starting from 90 days after discharge. The association between LOS and readmission risk has already been described in the literature ^[[32,34,35]]^, but to our knowledge, this is the only study that describes how the relationship evolves within 1 year and differentiates the effect of three different length of stay sub-groups.

In fact, Jencks [32] found a higher risk of re-hospitalization at 30 days (OR 1.27) with LOS higher (double) than expectations based on DRG (national refund system based on the main diagnosis), whilst Glans [35] and Low [36] documented higher re-hospitalization rate at 1 month with LOS longer than 5 days and 7 days, respectively.

The predictive role of selected laboratory values is already described in the literature [2,6,14,15,31,37]. Our study confirms and extends previously reported results of Dombrowski et al. [15] on the strong association between low albumin level and readmission at 1 month, since our observation interval was extended up to 1 year maintaining statistical significance.

An association between impaired renal function and readmission risk, was documented by Krumholz [6] and Muzzarelli [37] in selected populations of patients with heart failure, as well as by Zanocchi [2] in a mixed cohort of older adults, where creatinine ≥ 2.7 mg/dl was a predictor of unplanned readmissions, within a timeframe of 3 months of follow-up. In our study too, the probability of re-hospitalization increases along with a decrease in eGFR, with maximal effect in patients with eGFR < 40 ml/min (*p*-value = 0.002). Moreover, our study confirmed the strength of this relationship up to 1 year of follow-up and observed that the risk of readmission related to creatinine clearance remains similar for the groups with eGFR 40–60 and above 60 ml/min for the first 90 days after discharge and only starts to differentiate significantly thereafter. This may provide insights and inputs for further evaluation of informed follow-up schedules or tailored post-discharge programs.

In our study sample, the comorbidity burden, defined as CCI ≥ 6, was also found to be significantly associated with a higher probability of re-hospitalization at 1 year (*p*-value = 0.0001). The finding was expected and consistent with the literature. However, previous studies used a lower cut off of comorbidity (CCI ≥ 2) and were performed in a study population younger than ours.

Interestingly, we found that a value of systolic blood pressure ≤ 115 mmHg was associated with readmission at 1 year (*p*-value < 0.001). Only one previous study investigated systolic blood pressure as a predictor of re-hospitalization [37], and it found that the risk of re-hospitalization at 30 days in older patients (average age  77 years) was associated with systolic BP < 120 mmHg. It must be noted that this study was performed on patients with cardiovascular conditions, meanwhile in our study, the predictive value of low systolic blood pressure on readmission was investigated and confirmed in a heterogeneous population with a wide case mix. Our findings may reflect the impact of the severity of the condition at admission because low systolic blood pressure may be a clinical presentation sign not only of heart-related conditions but also of severe infections with hemodynamic instability. The negative predictive role of hypotension demonstrated in our study seems to confirm the observation of Benetos et al. [38], who found a negative relationship between low systolic blood pressure (< 130 mmHg), treatment with two or more antihypertensive drugs, and mortality. Low systolic blood pressure in older adults seems, therefore, to play a negative prognostic role on both mortality and readmission. Systolic blood pressure is systematically evaluated during admission in acute care settings, but its prognostic value in terms of readmission prediction might have been underestimated until now. According to our results, and similarly to the J-shaped relationship between systolic blood pressure and mortality already described in literature [39], low systolic blood pressure may offer a simple, promptly available and useful tool to predict readmission risk.

In our study, when the joint effects of selected prognostic variables were accounted for, LOS longer than 14 days, worse renal function, systolic blood pressure < 115 mmHg, and higher burden of comorbidity were independently associated with higher readmission risk.

Moreover, when we added diagnosis in the model, the above-mentioned variables were still significantly associated to the risk of re-admission. It is important to point out that the contribution of each of the four final variables was fairly similar and therefore these variables do not duplicate their prognostic significance. The overall performance of the final model was assessed calculating a “global” score for each subject.

The aim of this study was to evaluate the predictive role of the different components of CGA as well as of commonly utilized clinical variables, routinely assessed during clinical practice (systolic blood pressure, eGFR) on the risk of readmission. On the other hand, testing the predictiveness of validated clinical frailty scores was not among the aims of this study. However, when we added the electronic Frailty Index (Clegg et al. 2016) [40] to the significant variables in the final Cox model (namely length of stay, systolic blood pressure, eGFR, CCI), this index was not significantly associated with the hazard of readmission (data not shown). Such a result might further corroborate the strength of our model, since its prognostic value is independent of frailty (which is a well-known risk factor for worse outcome) when the effect of other variables is accounted for.

Three groups were identified when considering for the score two thresholds symmetrically above and below zero. As expected, there was a trend in the hazard for readmission for patients belonging to the three groups. Group 3 (with the highest scores) showed a hazard for readmission that was about 2.2 times that of group 1 (with the lowest scores), while group 2 (with the score centered on zero) showed a hazard for readmission that was about 1.5 times that of group 1. In absolute values, 134 out of 245 patients of group 3 were readmitted while 62 were expected; on the other hand, 112 out of 228 patients of group 2 were readmitted while 68 were expected. After 365 days of follow-up, the chances of readmission in the three groups were 0.369, 0.529 and 0.622, respectively.

In addition, to the best of our knowledge, this is the only study describing how readmission evolves over a timeframe of a whole year, taking into account the effect of risk factors.

It is possible that this model may provide highlights on the prognostic role of selected clinical variables and potentially inspire further research evaluating the respective and combined role of CGA components as predictors of readmission. We deem that this still remains a field of active interest in geriatric literature and clinical management, since, as already noted by Sun et al. [31], even if several scores have been proposed and validated in adults, an adequate index of re-hospitalization is still missing in older patients. Therefore, since our model is based on relevant routinely collected clinical parameters, it may offer useful and widely available insights to guide prognostic evaluation of the patients.

The strength of our study relies on the extensive CGA performed on the study population, which enabled the evaluation of separate and joint relations of each readmission parameter. CGA, implemented as part of routine geriatric assessment at admission, could be able to guide clinician’s decisions toward a patient-centered approach. Secondly, as further strength, CGA was performed extensively on our study population and very few CGA data are missing. Furthermore, the prospective design of the study, with active and structured follow-up over the post-discharge period, enabled us to capture all the readmissions that occurred, even if patients were readmitted to a different ward or a different hospital.

Moreover, the long observation period of our study, which was extended up to 365 days with multiple steps, along with contact schedules with patients and/or caregivers, provide innovative insights on the behavior of the relationships between the different variables over time.

Finally, our study shows that each of the parameters (i.e., LOS, low systolic pressure, eGFR and CCI) included in our last model should be carefully examined by physicians taking care of older patients as linked to greater risk of re-hospitalization. In particular, our study provides new evidence on the negative prognostic role of low systolic blood pressure in the elderly recommending its careful identification and amelioration.

Limitations should also be recognized, due to the observational design of the study, which does not establish cause–effect relationships. Moreover, the medium size of the study sample may not be sufficient enough to detect other valuable insights and, as such, further studies involving larger population may be required to confirm and extend our findings. Due to the characteristics of the acute geriatric setting and especially the high burden of comorbidity, it was not possible to conduct physical performance tests extensively.

Finally, the included patients came from a single ward in a single hospital, and this may limit the generalization of the results to different settings.

## Conclusions

In a geriatric population of multimorbid hospitalized patients, the re-hospitalization rate at 1 year was 46.1%.

Several predictors of readmission at 1 year were found, such as LOS > 14 days, albumin level < 30 g/l, GRF < 40 ml/min, systolic blood pressure < 115 mmHg, higher comorbidity (CCI ≥ 6), and main diagnosis of cardiovascular disease. When the joint effects of selected prognostic variables were accounted for, LOS longer than 14 days, worse renal function, systolic blood pressure < 115 mmHg, and higher burden of comorbidity remained independently associated with higher risk of readmission. Identifying a predictive role of low systolic blood pressure, may offer a simple, promptly available, and useful tool to predict readmission risk. Over a timeframe of 365 days of follow-up, the relationship between the above-mentioned variables with the risk of readmission is not constant but evolves with time, with some associations becoming significant only by prolonging the observation beyond the commonly utilized cut off of 30 days after discharge.

This study highlights the importance of performing an accurate CGA, since defined domains and variables contained in the CGA, when combined in model, may offer a tool to identify the most fragile patients with clinical and functional impairment enhancing their risk of unplanned early and late readmission.

## Data Availability

The data is available from the authors upon reasonable request.
